# Supplementary figures and images for: Multi-Omics-Based Analysis of the Effect of Longevity Genes on the Immune Relevance of Colorectal Cancer
Source: Biomedicines. 2025 Apr 30;13(5):1085. doi: 10.3390/biomedicines13051085 (PMC12109330; doi:10.3390/biomedicines13051085)

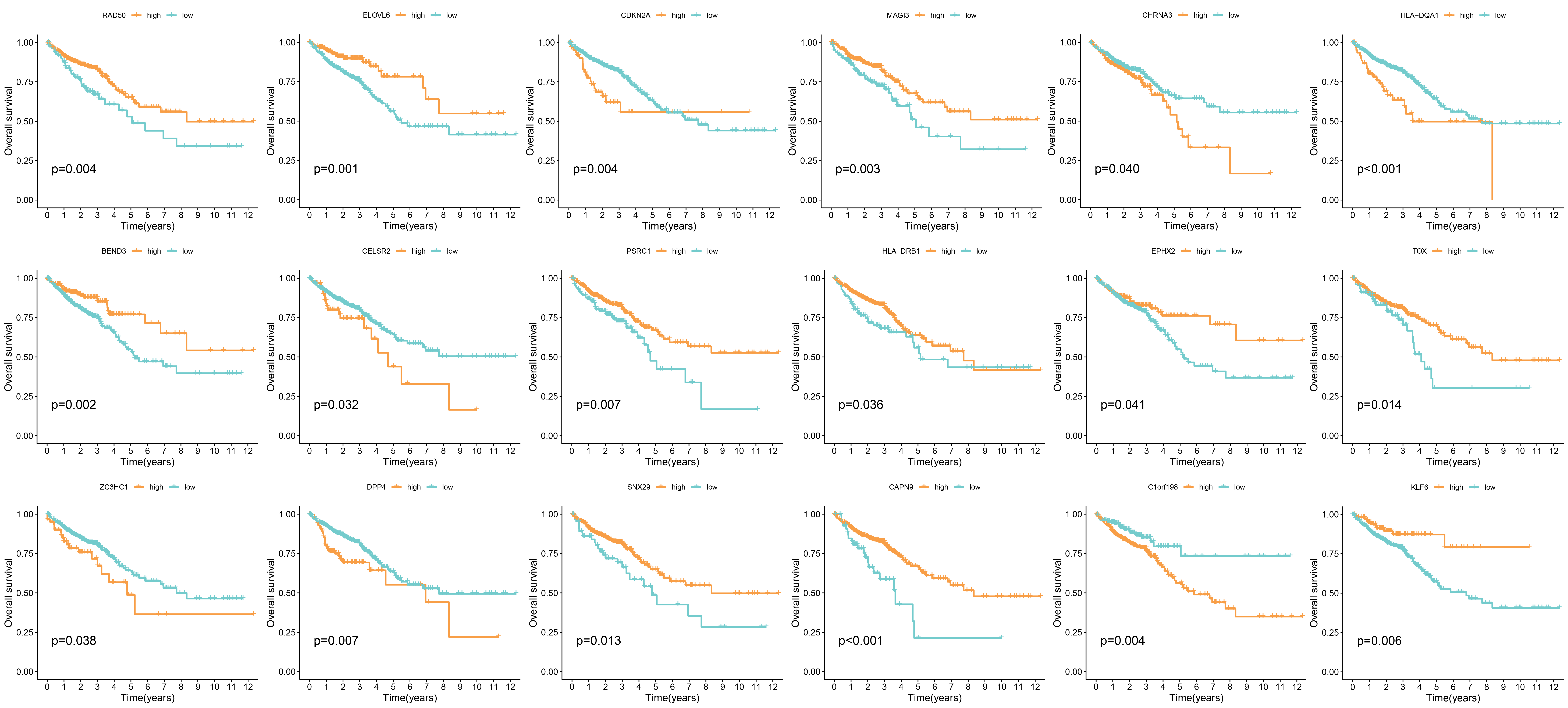

Supplement: Supplementary file 1 [file biomedicines-13-01085-s001.zip › S1.tif]

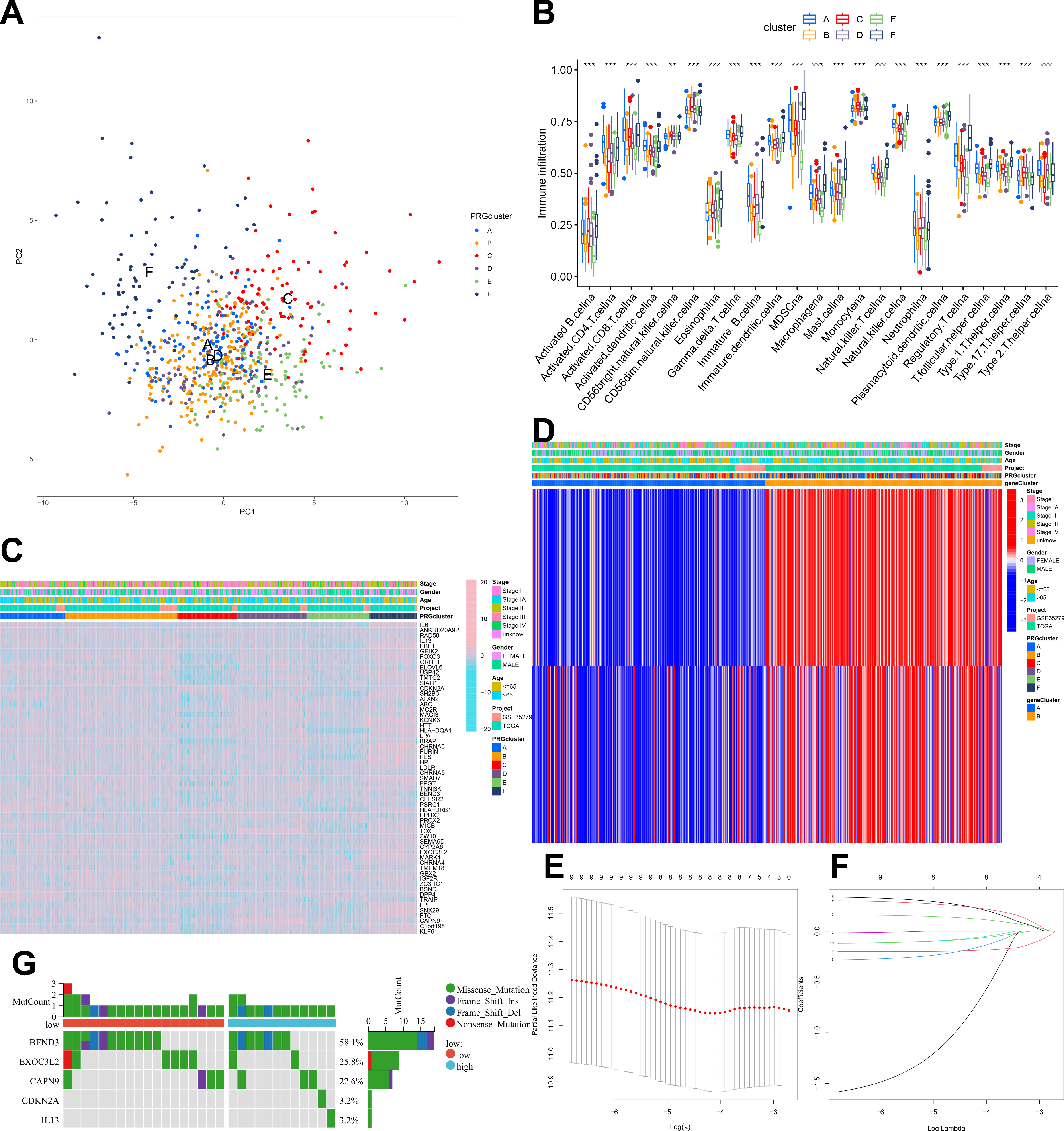

Supplement: Supplementary file 1 [file biomedicines-13-01085-s001.zip › S2.tif]
